# Suppression of MMP activity in bovine cartilage explants cultures has little if any effect on the release of aggrecanase-derived aggrecan fragments

**DOI:** 10.1186/1756-0500-2-259

**Published:** 2009-12-18

**Authors:** Bijue Wang, Pingping Chen, Anne-Christine Bay Jensen, Morten A Karsdal, Suzi H Madsen, Bodil-Cecilie Sondergaard, Qinlong Zheng, Per Qvist

**Affiliations:** 1 Nordic Bioscience A/S, Zhongguancun Life Science Park, Beijing 102206, PR China; 2 Nordic Bioscience A/S, Herlev Hovedgade 207, 2730 Herlev, Denmark

## Abstract

**Background:**

Progressive loss of articular cartilage is a central hallmark in many joint disease, however, the relative importance of individual proteolytic pathways leading to cartilage erosion is at present unknown. We therefore investigated the time-dependant release *ex vivo *of MMP- and aggrecanase-derived fragments of aggrecan and type II collagen into the supernatant of bovine cartilage explants cultures using neo-epitope specific immunoassays, and to associate the release of these fragments with the activity of proteolytic enzymes using inhibitors.

**Findings:**

Bovine cartilage explants were cultured in the presence or absence of the catabolic cytokines oncostatin M (OSM) and tumor necrosis factor alpha (TNFα). In parallel, explants were co-cultured with protease inhibitors such as GM6001, TIMP1, TIMP2 and TIMP3. Fragments released into the supernatant were determined using a range of neo-epitope specific immunoassays; (1) sandwich ^342^FFGVG-G2 ELISA, (2) competition NITEGE^373^ELISA (3) sandwich G1-NITEGE^373 ^ELISA (4) competition ^374^ARGSV ELISA, and (5) sandwich ^374^ARGSV-G2 ELISA all detecting aggrecan fragments, and (6) sandwich CTX-II ELISA, detecting C-telopeptides of type II collagen. We found that (1) aggrecanase-derived aggrecan fragments are released in the early (day 2-7) and mid phase (day 9-14) into the supernatant from bovine explants cultures stimulated with catabolic cytokines, (2) the release of NITEGE^373 ^neo-epitopes are delayed compared to the corresponding ^374^ARGSV fragments, (3) the MMP inhibitor GM6001 did not reduce the release of aggrecanase-derived fragment, but induced a further delay in the release of these fragments, and finally (4) the MMP-derived aggrecan and type II collagen fragments were released in the late phase (day 16-21) only.

**Conclusion:**

Our data support the model, that aggrecanases and MMPs act independently in the processing of the aggrecan molecules, and furthermore that suppression of MMP-activity had little if any effect on the quantity of aggrecanase-derived fragments released from explants cultures.

## Introduction

All though the pathogenesis joint diseases is not fully understood, major efforts have been allocated to the development of drugs aimed at down regulating proteases expression and acitivity involved in the degradation of the extracellular matrix of the joint. The protease repertoire of the chondrocytes is wide, and both aggrecanases, MMPs, and cathepsins have been associated with degradation and/or repair of the ECM of the articular cartilage in the joint [1-7].

To study the metabolic events leading to joint damage, cultures of articular cartilage has been a useful model system [5,8-12]. In bovine explants cultures stimulated with catabolic cytokines it has been demonstrated that both aggrecan and collagen fragments are released into the supernatant [5,13-15] and that inhibition of MMP-activity causes a suppression of both proteoglycan and type II collagen degradation [6]. However, release of aggrecanase-derived aggrecan fragments in explants cultures in the presence of protease-inhibitors have until now not been monitored by neo-epitope specific immunoassays.

The present study was initiated to develop and characterize new immunoassays for the quantitative detection of aggrecanase-derived aggrecan fragments carrying neo-epitopes in the interglobular domain of aggrecan, and to compare these tests to the MMP-derived aggrecan profile obtained from ex vivo cultures of bovine articular cartilage.

## Materials and methods

### Bovine articular cartilage explants

The stifle joints from young heifers were received from the local slaughterhouse, and cartilage explants were isolated and cultured essentially as described before [6], with protease inhibitor GM6001 used at 10 μM, and TIMP-1, TIMP-2, and TIMP-3 used at 50 ng/ml.

### Biochemical markers of aggrecan and collagen

#### a) Aggrecanase-derived aggrecan fragments carrying ^374^ARGSV (^374^ARGSV ELISA)

Monoclonal antibody 6D6 was developed by immunizing mice with synthetic peptide ARGSVILTVK-GGC conjugated to ovalbumin and performing fusion by standard techniques. MAb 6D6 was selected as it recognised the homologous sequence (ARGSVILTVK) but not the N-terminally elongated sequence GEARGSVILTVK.

A competition assay was designed by adding a ARGSVILTVK-GGC-biotin solution to streptavidin-coated microtitre plates. After washing three times, wells were incubated with 50 μL of supernatant prediluted in buffer and 100 μL prediluted 6D6 for 1 hour at 20°C with shaking. The plates were washed and incubated with peroxidase-conjugated, goat anti-mouse immunoglobulin, washed, and subsequently incubated for 15 minutes with TMB. The colour reaction was stopped and the absorbance was measured at 450 nm with 650 nm as reference.

#### b) Aggrecanase-derived aggrecan fragments carrying ^374^ARGSV and G2 (^374^ARGSV-G2 ELISA)

This sandwich assay was in principle as the ^374^ARGSV-G2 ELISA previously described [16] except that the capture antibody BC-3 was replaced with MAb 6D6.

#### c) Aggrecanase-derived aggrecan fragments carrying NITEGE^373 ^(NITEGE^373 ^ELISA)

Spleen cells from mice immunized with CPLPRNITEGE^373 ^conjugated in the N-terminus to KLH was used for fusion and development of monoclonal antibodies recognizing the aggrecan neo-epitope NITEGE^373^. MAb 1H11 was used for test development, as it did not recognize the elongated amino acid sequence PLPRNITEGEAR demonstrating specificity for the neo-epitope. A competition ELISA similar to the ^374^ARGSV ELISA described above was developed using MAb 1H11.

#### d) Aggrecanase-derived aggrecan fragments carrying NITEGE^373 ^and G1 (G1-NITEGE^373 ^ELISA)

Similar to the ^374^ARGSV-G2 ELISA described above except that biotinylated MAb 1H11 used as capture antibody.

#### e) MMP-derived aggrecan fragments carrying ^342^FFGVG and G2 (^342^FFGVG-G2 ELISA)

This immunoassay has previously been described [16].

#### f) MMP-derived type II collagen fragments carrying EKGPDP (CTX-II ELISA)

These collagen fragments were detected by the Serum PC CartiLaps ELISA [17] according to instructions of the manufacturer (IDS, UK).

## Results

### Tests for aggrecan fragments

To be able to monitor the degradation of the ECM and to link the release of closely associated protein fragments with the activity of certain proteases, it was decided to develop monoclonal antibodies to the neo-epitopes originating from the major aggrecanase site in aggrecan. Some of these neo-epitope specific antibodies have been described previously [16], but in the present study we report the development of MAb 1H11 recognising the neo-epitope NITEGE^373 ^and MAb 6D6 binding to ^374^ARGSV. Both neo-epitope sequences originate from the cleavage of aggrecan by aggrecanases at the amino acid sequence NITEGE^373-374^ARGSV.

**Figure 1:**
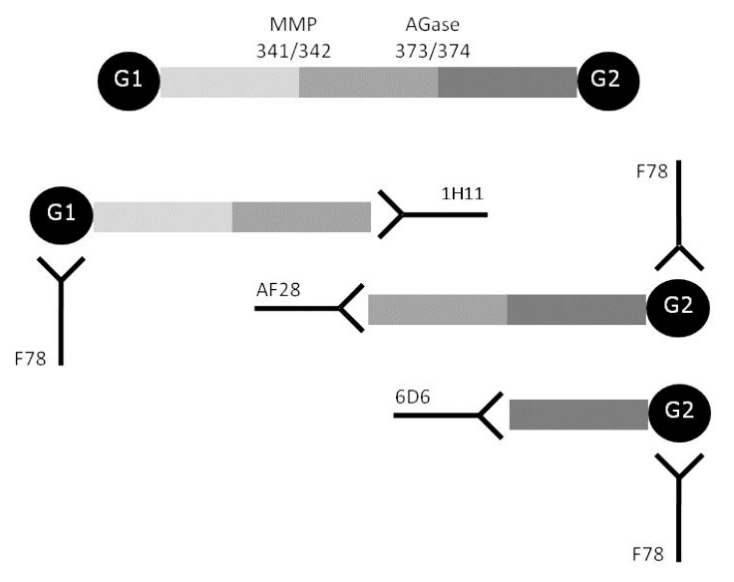
**Epitope-specificity of monoclonal antibodies binding to aggrecan fragments**. Schematic representation of the monoclonal antibodies selected for incorporation into immunoassays for quantification of aggrecan fragments carrying neo-epitopes. MAb F78 recognises a repeated epitope in the G1 and G2 domain, MAb AF28 binds to the MMP-derived neo-epitope ^342^FFGFG, MAb 1H11 binds the neo-epitope NITEGE^373^, and MAb 6D6 binds to ^374^ARGSV.

MAb 1H11 and MAb 6D6 were incorporated into competitive immunoassays as well as sandwich assays with MAb F78. The latter antibody recognized a repeated sequence in both the G1 and the G2 domain [16], thereby making it useful in sandwich constructions with antibodies binding to neo-epitopes in the interglobular domain (figure 1).

The basic characteristics of each (new) assay are summarized in table 1. It should be noted, that calibrator material was different in the competition and the sandwich assays, i.e. synthetic peptides vs ADAMTS-4 treated bovine aggrecan, and therefore absolute values cannot be compared.

**Table 1:** Summary of technical performance of four neo-epitope specific immunoassays

Parameter	NITEGE^373^	G1-NITEGE^373^	^374^ARGSV	^374^ARGSV-G2
Measuring range (ng/ml)	12.3 - 1000	120 - 4000	0.4 - 20	16.5 - 1000

Lower detection limit (ng/ml)	4.1	40.1	0.1	5.5

Intraassay variation (CV, %)	4.2 - 9.6	4.0 - 6.0	7.4 - 11.2	2.0 - 3.5

Interassay variation (CV, %)	7.1 - 13.2	7.5 - 13.4	9.9 - 12.4	8.9 - 10.3

Dilution recovery (mean; range,%)	103.6; 88.9 - 118.2	106.6; 94.6 - 119.2	101.4; 89.3 - 117.1	93.4; 82.7 - 103.5

Specificity (%)	PLPRNITEGE; 100 PLPRNITEGEAR; <0.1 ARGSVILTVK; <0.1		ARGSVILTVK; 100 GEARGSVILTVK; <0.1 PLPRNITEGE; <0.1	

The technical performance data was obtained by testing of supernatants originating from bovine cartilage explants cultures, and to support these data, bovine synovial fluid was tested in each of the tests. In bovine synovial fluid (n = 7), concentrations of the four analytes were; NITEGE^373^, 335-490 ng/ml; G1-NITEGE^373^, 371-866 μg/ml; ^374^ARGSV, <5 ng/ml; and ^374^ARGSV-G2, 23-49 μg/ml.

Subsequently, these immunoassays were included in a portfolio of neo-epitope specific immunoassays used for profiling supernatants of explants cultures.

#### Aggrecan and collagen fragments released from explants cultures with and without protease inhibitors

**Figure 2:**
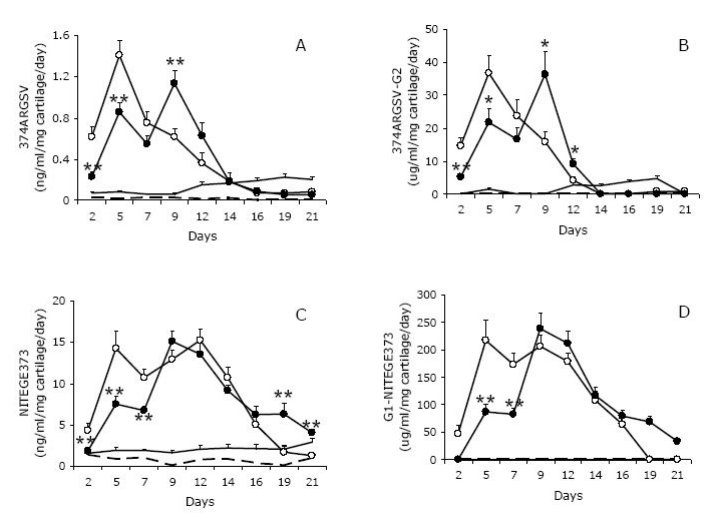
**Aggrecanase-derived aggrecan fragments released into the supernatant from cartilage explants stimulated with catabolic cytokines**. Bovine articular cartilage explants were stimulated with catabolic cytokines OSM and TNFα in the absence (open circles) and presence (closed circles) of the MMP-inhibitor GM6001. For comparison, parallel cultures were incubated with vehicle (continous line, no symbols) or metabolic inactivated at start by freeze-thaw cycles (dashed line). Supernatants were collected every second or third day and tested in competition ^374^ARGSV ELISA (figure 2A), sandwich ^374^ARGSV-G2 ELISA (figure 2B), competition NITEGE^373 ^ELISA (figure 2C) and sandwich G1-NITEGE^373 ^ELISA (figure 2D).

**Figure 3:**
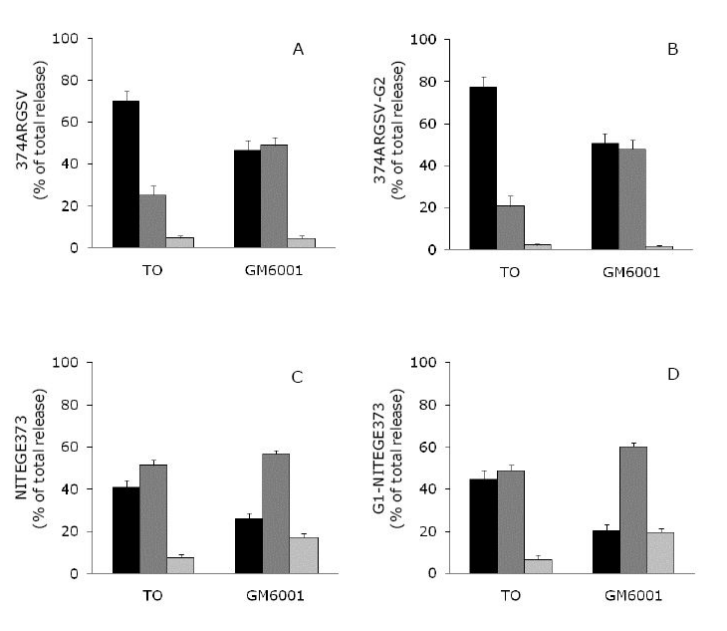
**Aggrecanase-derived aggrecan fragments released during the early, mid and late phase of cartilage explants stimulated with catabolic cytokines**. As for figure 2, but release of fragments in the early (day 2-7, black bar), mid phase (day 9-14, dark grey bar) and late phase (day 16-21, light grey bar) are expressed in percentage of accumulated release during the 21 day study period.

When bovine explants cultures were stimulated with OSM and TNFα, the release of aggrecan fragments carrying the neo-epitope ^374^ARGSV generated by aggrecanase activity were rapidly detected by both the ^374^ARGSV competitive ELISA (figure 2A) and the ^374^ARGSV sandwich ELISA (figure 2B). Quantitating and accumulating the release of fragments in the three different phases, i.e. early phase day 0-7, mid phase day 9-14, and late phase day 16-21, indicate that 70, 25, and 5% of the measured fragments are released in the early, mid, and late phase, respectively, in the ^374^ARGSV competitive ELISA (figure 3A). Co-incubation with the MMP-inhibitor GM6001 induced a delay in the release of these aggrecan fragments to 47, 49, and 4% of the fragments being detected by the ^374^ARGSV competitive ELISA (figure 3A) in the early, mid and late phase, respectively. The accumulated release of the fragments detected by the ^374^ARGSV competitive ELISA over the 21 days was 10.7-12.6% lower with inhibitor, which was not significant.

In contrast, the OSM/TNFα-stimulated release of fragments carrying the NITEGE^373 ^neo-epitopes extended into the mid phase, with a second peak around day 9-12 for the two assays (figure 2C and 2D). The GM6001 induced a further delay in release of the fragments carrying the NITEGE^373 ^neo-epitopes, and a clear single peak profile in mid phase was observed in these supernatants (figure 2C and 2D). Again, we did not observe significant changes in the accumulated release of fragments for this and the corresponding sandwich assay.

**Figure 4:**
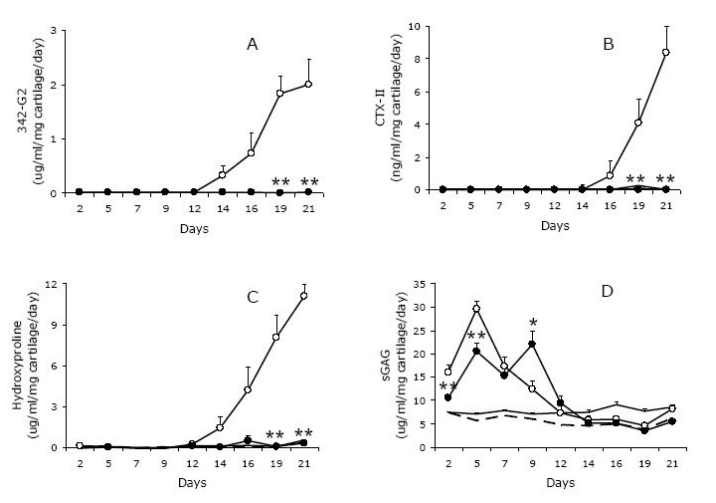
**Release of aggrecan- and type II collagen-derived fragments from cartilage explants stimulated with catabolic cytokines**. Supernatants from the experiment described in figure 2 was investigated in the sandwich ^342^FFGVG-G2 ELISA (figure 4A), the sandwich CTX-II ELISA measuring C-telopeptide fragments of type II collagen (figure 4B), sGAG (figure 4C) and hydroxyproline (figure 4D).

The cytokine stimulated release of MMP-derived fragments generated a completely different profile (figure 4A and 4B), and in addition, the release of these fragments remained undetected in the presence of GM6001.

At study termination alamar blue data confirmed cell viability in all explants cultures except metabolic inactivated (data not shown).

In parallel to these studies, the association between the release profile and the activity of other protease families were investigated by co-culturing the OSM and TNFα stimulated bovine explants in the presence of TIMP1, TIMP2, and TIMP3.

Across three experiments, none of these inhibitors induced a consistent suppression (or stimulation) of release of fragments into the supernatants (data not shown).

## Discussion

The study of metabolic processing of articular cartilage has traditionally relied on measurements of glycosamino glycans, hydroxyproline, isotope release etc [14], and recently neo-epitope specific antibodies and immunoassays have stimulated the research in this area [5,6,11,15,16,18,19]. To study the complex interaction of the MMPs and the aggrecanases in the degradation and repair of the extracellular matrix a broader portfolio of reliable analytical techniques is required.

We report here the development of four corresponding immunoassays all of which recognizes the neo-epitopes being generated by the aggrecanses at the NITEGE^373-374^ARGSV site. The application of these four tests allows the quantitative assessment of closely related pools of aggrecan fragments generated by aggrecanase, and furthermore we have compared the release-profile of these molecules to corresponding MMP-derived fragments.

### Aggrecan profiling in bovine explants supernatants

Under various experimental conditions up to nine different cleavage sites have been identified in the IGD, however, only two of these appear to occur in human tissue *in vivo*, i.e. the MMP-derived VDIPEN^341-342^FFGVG and the aggrecanase-derived NITEGE^373-374^ARGSV [7]. The characterization of the proteolytic degradation of cartilage by MMPs and aggrecanases was first described in the early 1990's [5,20-22], and since then, the relative importance of MMPs and aggrecanases in aggrecanolysis have been the subject of many studies.

Struglics and coworkers [23] identified at least nine different GAG-containing aggrecan fragments in human synovial fluid, with the ^342^FFGVG and ^374^ARGSV neo-epitopes contributing 80% of the total pool of aggrecan fragments. These and other observations led them to suggest that the degradation of aggrecan occur by at least two independent proteolytic pathways generating both ^342^FFGVG (the MMP-mediated) and ^374^ARGSV-fragments (the aggrecanase-mediated).

The data reported in the present study, which originates from a completely different experimental set up, is consistent with this model. Both types of neo-epitopes have been identified in the supernatants, however, their release is separated in time as we never find MMP- and aggrecanase-derived neo-epitope fragment in the same supernatant.

The delay in the release of the fragments carrying the NITEGE^373^neo-epitopes, compared to ^374^ARGSV fragments, i.e with 70% and 41% of the analytes released after the early phase, respectively, could be due to the anchoring of the aggrecan molecule through the G1 domain and the link protein to central hyaluronic acid filaments in the ECM [24]. Linking of the ^374^ARGSV neo-epitope to the ECM through a G1-HA/LP complex would be released through the action of MMPs, which would cleave at the VDIPEN^341-342^FFGVG intersection. We have seen no evidence of MMP activity (to the extent this produces neo-epitopes in aggrecan and type II collagen) and therefore is consistent with the linking of G1 to HA/LINK. Also, linking to ECM has not been associated with the other globular domains, i.e. G2 and G3.

### Further delays in release of aggrecanase-derived fragments induced by the MMP-inhibitor GM6001, but not overall reduction

Others have reported the application of neo-epitope specific immunoassays for quantitative detection of aggrecan fragments [5,16,25,26], however, this is the first report on the application of neo-epitope specific immunoassays for profiling of supernatants originating from explants cultured in the presence of protease-inhibitors.

First, it is important to establish, that GM6001 did not induce suppression of the overall release of the aggrecanase-derived aggrecan fragments, but merely delayed the release of these proteolytic fragments. The molecular mechanism behind this delay in the release of aggrecanase-derived fragments, which is influenced by MMP-activity, remains to be determined. However, it could be speculated that the effect of the MMPs in the early phase is indirect, i.e. does not generate detectable neo-epitopes in aggrecan and type II collagen, but act on other molecular structures which indirectly affects the release of these neo-epitopes into the supernatants.

It should be noted, that the explant model system, which requires molecular diffusion from inside of the explant to the surface of the cartilage surface, is highly dependent on the architecture of the tissue. Chondrocytes embedded in the cartilage will respond metabolically to cytokine stimulation and these responses will eventually determine the structural integrity of the tissue. Therefore, at the macroscopically level the structure of the explants is an important determinant of the release of fragments into the culture supernatants. In addition to MMPs, aggrecanases, and hyaluronidases, recent reports suggest m-calpain is important in mature bovine articular cartilage [27].

Surprisingly, we failed to detect a consistent effect of the aggrecanase inhibitors. Aggrecanase 1 (also called ATAMTS-4) and aggrecanase 2 (also called ADAMTS-5) are potent proteases capable of cleaving aggrecan at the 373-374 site [28], and we had expected at least TIMP3, which has been reported to inhibit the activity of both ADAMTS-4 and ADAMTS-5 [29,30], to suppress the release of the aggrecanase-derived fragments.

## Conclusion

We conclude that our data support the model that aggrecanases and MMPs act independently in the processing of the aggrecan molecules, and furthermore that suppression of MMP-activity have little if any effect on the quantity of aggrecanase-derived fragments released from explants cultures.

## List of abbreviations

ADAMTS: a disintegrin and metalloproteinase with thrombospondin motifs; BSA: bovine serum albumin; CTX-II: crosslinked C-telopeptide of type II collagen; DMEM: Dulbecco's modified Eagle's medium; ECM: extracellular matrix; ELISA: enzyme-linked immunosorbent assay; HA: hyaluronic acid; IMDM: Iscove's Modified Dulbecco's Medium; KLH: keyhole limpet hemocyanin; LP: link protein; MAb: monoclonal antibodies; MMP: matrix metalloproteinase; OA: osteoarthritis; OSM: oncostatin M; PBS: phosphase buffered saline; sGAG: sulphated gycosaminoglycan; TNF: tumour necrosis factor.

## Competing interests

All co-authors are full time employees of Nordic Bioscience. MAK and PQ are stock holders in Nordic Bioscience. No other competing interests.

## Authors' contributions

BW carried out antibody development, immunoassay development, data interpretation and participated in manuscript preparation. PC carried out explant cultures and supernatant profiling. ACBJ participated in immunoassay development and data interpretation. MAK participated in design of the studies, data interpretation and manuscript preparation. SHM and BCS participated in data interpretation and manuscript preparation. Qinlong Zheng participated in antibody development, explants cultures, and supervised part of the research. Per Qvist participated in study design, data interpretation, and carried out the drafting of the manuscript. All authors have read and approved the final manuscript.
